# An exploratory survey measuring stigma and discrimination experienced by people living with HIV/AIDS in South Africa: the People Living with HIV Stigma Index

**DOI:** 10.1186/1471-2458-14-80

**Published:** 2014-01-27

**Authors:** Monika ML dos Santos, Pieter Kruger, Shaun E Mellors, Gustaaf Wolvaardt, Elna van der Ryst

**Affiliations:** 1 Psychology Department, University of South Africa, PO Box 392, 0004 Pretoria, South Africa; 2 International HIV/AIDS Alliance, Hove, UK; 3 Foundation for Professional Development (FPD), Pretoria, South Africa; 4 Independent Consultant, The Research Network, Sandwich, UK

**Keywords:** HIV/AIDS, Stigma, Discrimination, South African context

## Abstract

**Background:**

The continued presence of stigma and its persistence even in areas where HIV prevalence is high makes it an extraordinarily important, yet difficult, issue to eradicate. The study aimed to assess current and emerging HIV/AIDS stigma and discrimination trends in South Africa as experienced by people living with HIV/AIDS (PLHIV).

**Methods:**

The PLHIV Stigma Index, a questionnaire that measures and detects changing trends in relation to stigma and discrimination experienced by PLHIV, was used as the survey tool. The study was conducted in 10 clinics in four provinces supported by the Foundation for Professional Development (FPD), with an interview total of 486 PLHIV. A cross-sectional design was implemented in the study, and both descriptive and inferential analysis was conducted on the data.

**Results:**

Findings suggest that PLHIV in this population experience significant levels of stigma and discrimination that negatively impact on their health, working and family life, as well as their access to health services. Internalised stigma was prominent, with many participants blaming themselves for their status.

**Conclusion:**

The findings can be used to develop and inform programmes and interventions to reduce stigma experienced by PLHIV. The current measures for dealing with stigma should be expanded to incorporate the issues related to health, education and discrimination experienced in the workplace, that were highlighted by the study.

## Background

Stigma, defined as a mark of disgrace associated with a particular circumstance, quality or person, is not new to public health, nor is it unique to HIV/AIDS [1]. History provides a number of examples of prejudice and discrimination against people with specific diseases [2-5]. Even after the germ theory of disease became widely accepted, negative attitudes towards certain diseases and patient groups continued, for example, some persons with syphilis were ‘innocent’; others were not. Often, even physicians were reluctant to treat patients from certain groups, considering them not worthy [5].

Stigma interferes with HIV prevention, diagnosis and treatment and can become internalised by people living with HIV/AIDS (hereafter referred to as ‘PLHIV’) [6]. Importantly, stigma is often enacted through discrimination (defined as the rejection or prejudicial treatment of different categories of people or things, especially on the grounds of race, age, health status or gender), hostility and prejudice against PLHIV (as well as their partners and families), denying them equal access to essential services in many cases [4]. A study by Holzemer et al. [7] on a sample of 726 participants, demonstrated that perceived HIV stigma has a significant negative impact upon quality of life of PLHIV. A descriptive study conducted in several countries indicated that coping mechanisms in PLHIV appears to be self-taught and not supportive in terms with dealing with perceived stigma [8].

Stigmatisation associated with HIV/AIDS is underpinned by many factors, such as lack of understanding of the disease (including misconceptions about modes of transmission), lack of access to treatment, irresponsible media reporting, the incurability of AIDS, prejudice and fears relating to a number of socially sensitive issues (including sexuality, disease and death, and drug use) [9,10]. Not only is HIV/AIDS-related discrimination a human rights violation, but it is also necessary to address such discrimination and stigma in order for public health goals related to HIV/AIDS prevention and management to be achieved [10].

HIV/AIDS related stigma appears to be declining in South Africa, as shown by comparing the findings of the 2002 National HIV and AIDS Household Survey with the 2005 survey. The 2002 survey indicated that 80.8% of participants would not sleep in the same room as someone who was HIV positive, while 94.5% would not talk to someone who was HIV positive [11]. In comparison, data from the 2005 survey indicated that less than half of participants (46.5%) indicated hesitance about marrying someone with HIV/AIDS, while 46.8% said they would have a problem having protected sex with a partner who has HIV/AIDS [12].

Notwithstanding, HIV/AIDS is still associated with significant stigma in Southern Africa [13]. The advances made by scientists in the diagnosis, prevention and treatment have unfortunately not been matched by advances in social acceptance of the disease. The influence of cultural and traditional dogma is still very powerful in Africa and is also heavily permeated by religion, which can add a further burden against judgment, stigma and discrimination against PLHIV [8,13].

Within the healthcare sector in a number of African countries, PLHIV have reported extensive neglect, as well as verbal and physical abuse. These occurrences have also been observed by nurses caring for them [14]. A study on culture, women’s rights and HIV in Namibia, undertaken by the Southern Africa HIV/AIDS Information Dissemination Services (SAFAIDS), confirmed the low status of women in Namibia’s rural areas, which is reinforced by cultural heritage and feed stigma, since women carry the blame for many things among them the spread of HIV. According to these researchers, women are not free to speak of their HIV status to their partners for fear of violence [15]. Furthermore, stigma appears to be more severe for women than men [10,16,17]. Exclusion of and discrimination towards PLHIV is one of the consequences of stigma, and it may force people who are infected to hide their HIV serostatus, and in many cases, to continue engaging in high-risk behaviour [8,18].

The People Living with HIV Stigma Index is a community research and advocacy initiative that has been developed by and for PLHIV [19]. It is the result of a partnership between the International Planned Parenthood Federation (IPPF), UNAIDS, the International Community of Women living with HIV (ICW) and the Global Network of People Living with HIV (GNP+). Since 2004 these partners have led a broad consultation process which resulted in the final comprehensive tools, built on the existing work by numerous organisations and specialists in index design [20].

The Index was developed specifically to evaluate efforts to address stigma and discrimination related to PLHIV and to build an evidence base to make it possible to compare data across countries, through provision of a tool that measures and detects changing trends in relation to stigma and discrimination experienced by PLHIV. It aims to combine research with empowerment by placing PLHIVs at the centre of the process as interviewers, interviewees and as local users of the information generated [19]. The Index further aims to inform the development and implementation of national policies that protect the rights of PLHIV, and shape the design of programmatic interventions so that they consider the issue of HIV/AIDS related stigma and discrimination within their context [20]. It also aims to address stigma relating to HIV/AIDS whilst advocating on the key barriers and issues perpetuating stigma. It is designed to document how people have experienced - and been able to challenge and overcome - stigma and discrimination relating to HIV/AIDS [20].

The primary research tool utilised in the PLHIV Stigma Index is a questionnaire that is divided into three main sections, covering perceptions of self and internal stigma as well as examples of stigma or discrimination in different settings such as the home, community, workplace, religious or healthcare settings. The questionnaire also captures demographic information pertaining to participants; experiences of stigma and discrimination from other people; access to work, health and education services; internalised stigma; rights, laws and policies; effecting change; testing/diagnosis, disclosure and confidentiality; HIV treatment and care; having children; and lastly, problems and challenges experienced.

The questionnaire was piloted in 5 countries in 2006 (India, Lesotho, Kenya, South Africa, Trinidad and Tobago). It was finalised in 2008 and the first country rollout was in the Dominican Republic. The initiative was announced at the XVII International AIDS Conference in 2008 [21]. In 2013 it was announced that the programme had been rolled out in more than fifty countries [22]. A number of reports based on this initiative detailing the extent of stigmatisation due to HIV/AIDS have subsequently been released, including studies in South Africa (2012), Ethiopia (2011), the Asia-Pacific region (2011) reflecting data from in nine countries in Asia and the Pacific (Bangladesh, Cambodia, China, Fiji, Myanmar, Pakistan, Philippines, Sri Lanka, Thailand), Malaysia (2012), Laos (2012) and other countries (reports of completed studies are available on the PLHIV Stigma Index website) [22].

Against this background, the present study using the PLHIV Stigma Index was conducted to gain more insight into the way that stigma and discrimination manifests among PLHIV in South Africa to add to the growing international database reflecting the extent of stigma and discrimination experienced by PLHIV, and to determine whether stigma can be tied to specific demographic indicators.

## Methods

The PLHIV Stigma Index was used as the instrument in this study. Small adaptations were made to the Index, including the quantifying of all qualitative responses into nominal and ordinal scales and the inclusion of South Africa’s best-known national law and policy guidelines as per the Index directives. The original open ended questions at the end of the original Stigma Index (Section 3E – Problems and Challenges) were assigned response categories to enable quantitative analysis of the responses, by providing a list of possible answers for each question in this subsection; for example, the question ‘What do you see as the main problems and challenges in relation to testing and diagnosis?’ were provided with the categorical options of *accessibility, confidentiality, counselling* and *resources*. The response options for all the questions in this subsection were decided upon after consulting with HIV experts, and were implemented in order to facilitate the quantifying of information, data coding and analysis.

### Study population and design

The study was conducted in ten HIV clinics supported by the Foundation for Professional Development (FPD). The clinics are located in four provinces; Gauteng (3); North West (3); Mpumalanga (2) and Limpopo (2). The PLHIV attending FPD-supported clinics were selected based on access and availability. Convenience sampling was used as the study was limited to FPD clinics and only participants who had voluntarily disclosed their HIV positive status could be involved for ethical reasons. Participants who were current patients at the clinics and with whom fieldworkers were already familiar were recruited. This conforms to the peer-based model as recommended by the manual of the PLHIV Stigma Index [20].

A target sample size of 500 was determined by the resources available and time constraints, and was not based on statistical calculations. It was decided that this would provide an adequate number of participants to allow for meaningful exploratory insights and conclusions to be drawn.

In order to address language barriers that may confound the results, the Index was translated from English into three indigenous South African languages (Zulu, Sotho and Venda) by linguistic experts.

### Ethical considerations

Participation was voluntarily, and no patient was excluded based on gender, ethnicity or socio-economic status. The interviewers ensured that all participants provided their written informed consent on the Patient Information and Informed Consent form before starting the interview. Their right not to participate, as well as to withdraw from the study at any point, was emphasised. It was deemed particularly important to do so in this study as the data collected was of a personal nature and also focused on sensitive issues such as sexual relationships and instances of discrimination. Only participants above the age of 18 were interviewed, and only participant who were considered competent to give informed consent were included. Every effort was made to avoid interviewing those with medically documented severe mental health disabilities (such as dementia or psychosis) that could have impaired their ability to provide informed consent.

Data collected was kept confidential. The questionnaire used in the survey was designed to help ensure confidentiality, and individual questionnaires were identified only by a unique identifying code. Confidentially was further enhanced by making use of a small group of FPD employed HIV-positive interviewers, as PLHIV are generally best placed to know and understand the problems that might be caused by any leakage of information about their peers. The aspect of creating an appropriate boundary (such as participant confidentiality) between being in the interview process, and other contact that the interviewer and interviewee might have as members of the same community, was emphasised during fieldwork training. All interviewers and team leaders also signed confidentiality agreements. Those responsible for data capturing, cleaning and analysis did not have access to personal details of the interviewees or the informed consent form.

Written approval for the study was obtained from the South African Medical Association Research Ethics Committee (SAMAREC) in September 2010, Department of Health (DoH) Trial Number: DOH-27-1110-3057.

### Fieldworker training

In the spirit of participatory research advocated by the PLHIV Stigma Index, field workers were recruited from staff based at various clinic sites (primarily lay counsellors) to conduct the interviews. These interviewers were trained to guide participants through completion of the questionnaire in individual interviews about their experiences of stigma and discrimination, thus facilitating empowerment and trust between interviewer and interviewee [22].

All interviewers underwent an intensive two day training programme, conducted by the study investigators, prior to the commencing of the interviews. Training included aspects of how to conduct an interview, good clinical practice, including the importance of informed consent and confidentiality, defining of key concepts and the process for completing the questionnaire. As the interviewers were all FPD employed staff from the ARV clinics they were already familiar with indicators of when referral to support service was necessitated. This aspect of the interview process was also covered extensively during training.

### Interview procedure

Participants were fully informed as to the nature of the study, and were assured of confidentiality during the entire study process. The process of filling in the questionnaire was shared with the participants in an individual interview. Specific concepts, such as ‘stigma’ and ‘discrimination’ were explained and definitions of the words were provided to the participants. The study adhered to a spirit of participatory research and of equal power and respect between the interviewer and participant, for example, by training interviewers to sit next to participants and to strive to make the experience an empowering one for the participant. All participants were thanked for their participation in the study and were referred to appropriate local organisations if the interviewer determined that they required specific support after the interview.

### Data analysis

In an effort to improve the utility of the questionnaire as a measurement of the intensity of stigma, a scale was constructed to measure *stigma and discrimination.* The scale was constructed by adding those items of the questionnaire where stigma or discrimination were attributed by a participant specifically to their HIV status together in a linear (unweighted) fashion, thus producing a stigma/discrimination scale in the range of 1 – 11 (the variables that were included are those which refer to stigma/discrimination, as listed later on in Table 1 below). The relationship of these scales to various demographic measurements was explored with the use of regression analyses, and where present, relationships were further investigated with appropriate statistical techniques such as for example, t-tests and analyses of variance.

**Table 1:** **Experience of stigma and discrimination**^**a**^

**N=486**	**Never experienced (%)**	**Experienced (Once to often) (%)**	**Not indicated (%)**
Being gossiped about	215 (44.2)	254 (52.3)	17 (3.5)
Verbally insulted/harassed, threatened	339 (69.8)	138 (28.3)	9 (1.9)
Husband/spouse/other household member have been discriminated against	358 (73.7)	116 (23.8)	12 (2.5)
Sexual rejection	368 (75.7)	101 (20.8)	17 (3.5)
Manipulation by partner	373 (96.7)	101 (20.8)	12 (2.5)
Excluded from social gatherings	399 (80)	91 (18.8)	6 (1.2)
Physically assaulted	400 (82.3)	78 (16.1)	8 (1.6)
Excluded from family activities	406 (83.5)	75 (15.5)	5 (1)
Physically harassed	387 (79.6)	75 (15.5)	24 (4.9)
Discriminated against by other PLHIV	404 (83.1)	71 (14.6)	11 (2.3)
Excluded from religious activities	417 (85.8)	50 (10.3)	19 (3.9)

^a^Questions relate to the previous 12 months.

All raw data was coded and captured in a spread sheet for analysis. Data analysis was conducted with the use of the STATISTICA suite of statistics software [23,24]. Descriptive statistics and frequencies were calculated for various variables. Where indications of stigma or discrimination were found, relevant statistical measures were used to relate this to other categorical variables, where appropriate.

## Results

### Demographic data

A total of 486 records of PLHIV who participated in the study were included in the analysis. This is less than the 500 participants originally aimed for due to one of the fieldworkers being unable to proceed with interviews.

The demographic characteristics of the 486 patients are reflected in Table 2. More than half (60.5%) of participants were sexually active at the time of participating in the study, while 14.2% of participants indicated that they suffered from some form of physical disability. Nearly three quarters of participants (71.3%) indicated that they did not belong to any of the traditional HIV/AIDS high-risk groups. Few participants self-identified as gay or lesbian (0.8%) (however, they may have chosen the category ‘men who have sex with men’ and vice versa), sex workers (0.4%), intravenous drug users (1.2%), refugees (1.9%), internally displaced (2.5%), migrant workers (1.2%) or prisoners (1.4%). A few male participants were in a ‘men who have sex with men’ relationship (2.3%), while none belonged to transgender groups.

**Table 2:** Demographic characteristics of sample

**Variable**	**Category**	**Number (%) N=486**
***Province***	Gauteng	252 (51.9)
	Limpopo	154 (31.7)
	North West	54 (11.1)
	Mpumalanga	28 (5.3)
***Gender***	Male	125 (26.7)
	Female	356 (73.3)
***Age group (years)***	15–24	30 (6.17)
	25–39	285 (58.6)
	40–49	123 (25.3)
	≥50	45 (9.3)
***Years living with HIV***	<1	110 (22.6)
	1–4	215 (44.2)
	5–9	118 (24.3)
	≥10	34 (7.0)
***Education***	No formal education	17 (3.5)
	Primary school	73 (15)
	Secondary school	312 (64.2)
	Technical College/University	80 (16.5)
***Household income***	≤R1000	171 (35.2)
	R1001-R3000	143 (29.4)
	R3001-R5000	55 (11.3)
	R5001-R10000	30 (6.2)
	≥R10001	19 (3.9)

Unemployment was high, with 51.4% of participants indicating that they were unemployed. As would be expected, poverty levels were high, with less than half of households having a monthly income of >R3 000. More than half of these households (53.09%) did not have enough food for between 1 and 4 days in the past month.

Approximately half of the participants lived in a small town or village (50.6%), while 27.8% lived in a rural village and 20% lived in large towns or cities (for an indication of South African population demographics/poverty levels in the respective provinces, please see http://www.statssa.gov.za/publications/Report-03-10-03/Report-03-10-032009.pdf) [25]. On average about 3.69 persons lived in each household, of which 1.07 were children younger than 14 years old and 0.3 were people older than 50 years of age. The majority of households (85.4%) had no children or youths living as AIDS orphans. However, 6.4% of households had one AIDS orphan, 3.1% two, and 4% three or more orphans.

### Experience of stigma and discrimination from other people

Participants were asked to indicate whether they ever experienced one or more incidents of stigma or discrimination from other people.

Data in Table 1 demonstrates that participants experienced significant levels of stigma and discrimination, with a belief that they were gossiped about being the most common. Other common forms of stigma and discrimination included verbal insults, discrimination against husband/spouse/other household member, psychological manipulation by partners and sexual rejection. Of the 16.1% who indicated that they had been physically assaulted, 57.7% indicated that the assault was perpetrated by a husband, wife or partner. Those that were physically assaulted by a partner were more likely to have a disability (the effect is significant; χ^*2*^=17.249, p=0.002). The lowest level of stigma and discrimination were related to their religious activities.

The participants’ perceptions of the reasons why they believed they were victims of stigmatisation and the results are summarised in Table 3.

**Table 3:** Perceived reasons for stigma and discrimination experienced

**N=486**	**n**	**HIV status (%)**	**Other (%)**	**HIV and other (%)**	**Not sure (%)**
Being gossiped about	254	142 (55.9)	13 (5.1)	57 (22.4)	24 (9.4)
Verbally insulted/harass/threatened	138	86 (62.3)	19 (13.8)	28 (20.3)	2 (1.4)
Excluded from social gatherings	101	35 (34.7)	26 (25.7)	22 (21.8)	10 (9.9)
Physically assaulted	78	40 (51.3)	17 (21.8)	11 (14.1)	8 (10.3)
Excluded from family activities	75	25 (33.3)	31 (41.3)	8 (10.7)	4 (5.3)
Physically harassed	75	41 (54.7)	17 (22.7)	11 (14.7)	4 (5.3)
Excluded from religious activities	50	4 (8.0)	32 (64.0)	6 (12.0)	1 (2.0)

These findings suggest that those experiencing gossiping and verbal insults are most likely to attribute it to their HIV status while exclusion from religious activities are the least related to be attributed to their HIV status.

A multivariate regression analysis was conducted to determine the extent to which participants’ scores on the composite stigma/discrimination scale (described above) could be predicted by demographic variables. Independent variables entered into the model were those listed in Table 2 in addition to: *relationship status; length of time involved with partners; whether the respondents are sexually active; whether they suffer from any disabilities; availability of food; province in South Africa where they live; type of area in which they stay;* and *number of people in their respective households*. Categorical (nominal level) variables were dummy coded for inclusion in the model.

A significant effect was found [F(22,164)=4.277 p<0.0001] with a multiple correlation coefficient of R=.604, which implies that about 36.5% of the variance in the stigma and discrimination variable could be explained by demographical variables. When the contribution of each individual variable to the regression model was considered, it was found that the variables which make a significant contribution to the model are *age group* (t=-2.118; p=0.0357), *disability* (t=-3.517; p=0.0006), *province* (t=-3.197; p=0.0017) and whether the participant was *living in an urban, town/village* or *rural area* (t=2.263; p=0.0250).

Further analysis of these significant variables showed that if age group is correlated with the stigma/discrimination scale on its own, a modest correlation could be found, of r=– 0.1118, which was nonetheless significant (p=0.026), which implies that age is correlated with stigma/discrimination to a modest but significant degree. A *t*-test showed that experience of stigma/discrimination is affected by whether a participant suffers from a disability to a highly significant degree (t=-7.912; p<0.0001). Participants who had a physical disability experienced incidents of stigmatisation or discrimination to a significantly lesser degree. The group means for the disability and non-disability groups were found to be 3.967 and 1.578 respectively and an effect size of Cohen’s d=1.028 was found.

To test the influence of province on stigma/discrimination scale, data from only two of the provinces could be used. This is due to there being no participants from any urban area at all in the sample from Mpumalanga and only 6 participants from areas designated as town or village, and only 2 urban participants from the North West Province. This left a comparison between Gauteng and Limpopo, respectively the wealthiest and the poorest provinces in South Africa [25].

A two-way analysis of variance using Province (Gauteng and Limpopo) and area of residence (rural, small town or village or rural area) found a significant effect for province (F=14.301; p<0.0002) and area (F=9.947; p<0.0001) as well as a significant but modest interaction effect (F=3.989; p=0.0194). Post hoc Sheffé tests showed that rural participants from the Gauteng province differed significantly from all the other sets of participants. Table 4 shows mean scores and p-values of rural participants from Gauteng and the p-values of comparisons of this group with other categories in the analysis.

**Table 4:** Mean stigma scores for Gauteng and Limpopo by area (Rural/Town/Urban)

**Cross classification**	**Mean stigma score**	**p-value in post hoc Sheffe comparisons**
Gauteng/rural	3.938	-
Gauteng/town or village	1.795	0.0001
Gauteng/urban	2.234	0.0105
Limpopo/rural	2.026	0.0124
Limpopo/town or village	0.579	0.0000
Limpopo/urban	1.059	0.0016

The reason why participants from rural areas in Gauteng should experience higher levels of stigma/discrimination than any of the other areas is not known. A further analysis was performed to determine whether this could be linked to employment status, but this did not yield any significant results.

### Internalised stigma

Indications of internalised stigma are summarised in Table 5. These data indicate that PLHIV in this study experience high levels of internalised stigma.

**Table 5:** Internalised stigma

	**N (%)**	**Missing (%)**
Blames self	239 (49.2)	14 (2.9)
Feels ashamed	231 (47.5)	20 (4.1)
Feels guilty	199 (40.9)	19 (3.9)
Have low self esteem	155 (31.9)	22 (4.5)
Blame others	109 (22.4)	24 (4.9)
Feels suicidal	72 (14.8)	21 (4.3)
Feel they should be punished	49 (10.08)	23 (4.7)

A large percentage of participants decided not to have more children because of their HIV status (60.1%); which can also be attributed to internalised stigma. Slightly less than a third respectively decided not to get married or to have sex (30% and 27%). Of concern is the high percentage of participants who isolated themselves from their friends and family or from social gatherings (16.9% and 14% respectively). The percentage of participants who opted to avoid going to clinics when needed was 14.4%, while 8% would avoid going to hospital. Just less than 10% (9.7%) of participants had stopped working due to their experienced internalised stigma, while 7.2% had not applied for jobs or promotions, and 4.4% withdrew from education and training.

More than half of the participants indicated that they have a fear of being gossiped about (54.1%), while about 26.7% feared verbal insults and harassment. Of major concern is the 18.1% of participants that feared physical harassment and the 17.5% of participants that feared actual physical assaults. Approximately 36.6% of participants were afraid that someone would not want to be sexually intimate with them due to their HIV status.

As in the case of stigma and discrimination above, an *internalised stigma* scale was produced from a linear combination of items reflecting instances of internalised stigma (using the variables from Table 5, above). This produced a scale with a range of 1 – 7. A regression analysis was performed to determine the effect of various demographic variables on internalised stigma, using the same demographic variable as those listed above in reference to the sigma/discrimination scale. A significant effect was found (F (22,177)=1.765; p=0.0234) with a multiple correlation of R=0.424, which means that only about 18% of the variance in internalised stigma could be explained. The only variables to make a significant contribution to the model were *age category* (t=-2.497; p=0.0134) and *relationship status* (t=-2.742; p=0.0067).

Taken on its own, the correlation between age group and internalised stigma was not significant. An one-way analysis of variance could not find a significant relationship between the different categories of relationship status (i.e. married and living together, married but living separately, in a relationship but living separately, divorced or separated, single or widow/widower) and internalised stigma (F=1.557; p=0.1709).

### Access to work, health services and education services

The HIV status of participants negatively impacted on their working life, as well as their access to health and education services. Fifty-seven (11.7%) participants indicated that they had lost their job in the past 12 months, of these more than half indicated it was at least partially due to their HIV status (due to discrimination and/or being too sick to work). Additionally, 7.7% of participants indicated that they had been refused an employment opportunity due to their HIV status. Approximately a tenth of participants (9.3%) were forced to change their accommodation or were unable to rent accommodation in the past 12 months, of these more than half felt it was at least partly due to their HIV status. Very few participants indicated that they or their children have been excluded from educational institutions.

Almost a third of participants (31.5%) indicated that they were satisfied with their health at the time of participating in the study. Twenty-six of 470 (5.5%) of participants indicated that they had been denied access to health services due to their HIV status in the past 12 months, while 6.4% (26/405) indicated they had been denied access to family planning services and 10.5% to sexual and reproductive health services.

### Knowledge and experience of rights, laws and policies

Just less than half of the participants were not aware of the Declaration of Commitment on HIV/AIDS (48.8%), or the AIDS Charter (49.2%). Among those that were aware of the Declaration of Commitment on HIV/AIDS and the AIDS Charter, 65.3% of participants had read and/or discussed the Declaration of Commitment on HIV/AIDS while even more had read and/or discussed the AIDS Charter (73.4%).

Relatively small proportions of participants experienced violations of their rights. The most common violations were being forced to submit to a medical or healthcare procedure (6.6%) and being denied health insurance (5.3%). Of those that have experienced violation of their rights, more than half (54.7) had tried to obtain legal redress, of these, 21.3% indicated that nothing had happened to resolve the situation. Three main reasons contributed to people’s choice for not taking legal redress; namely fear of intimidation (33.3%), no or little confidence in the outcome (19.4%), and financial concerns (16.7%).

A number of participants (29.8%) indicated that they had tried to mobilise government employees to take action against violation of rights, while slightly less (26.6%) went to local and national politicians to take action against such abuses. About two thirds of participants felt that the actions by government employees or politicians had actually dealt with the matter satisfactorily.

### Effecting change

Sixty-one point 6 percent of participants were aware of an organisation or group that could assist them if they experienced stigma or discrimination from others. Most participants (90.6%) were aware of HIV support groups. However, only 4.7% were aware of non-governmental organisations. Though participants tended to be aware of these groups or organisations, only 35.2% had sought help from any of the above groups to resolve issues of stigma and discrimination.

The following four aspects were highlighted by the participants as the most important aspects to address stigma: providing support (emotional, physical and referral) (28.6%), educating people living with HIV (25.7%), advocating for the rights of all PLHIV (23.3%), public awareness and knowledge among the public (17.5%). However, only 1% voiced support for advocating the rights of marginalised groups.

### Disclosure and confidentiality

Participants to a large extent disclosed their HIV status to healthcare workers, other PLHIV, spouses and other adult family members themselves. In general, the closer the relationship, the more willing participants living with HIV were to inform others of their HIV status. On the other hand, fewer participants informed their community leaders, teachers or other government officials, and employers about their HIV status themselves. In fact, the majority of participants indicated that these groups were not aware of their HIV status. Few participants indicated that their HIV status was disclosed without their consent.

More than 70% of participants indicated that they did not feel under pressure to disclose their HIV status by either PLHIVs or other individuals or groups. Forty-one (8.4%) participants indicated that a healthcare professional had told other people about their HIV status without consent, while 72.8% stated that this had never happened to them. A number of participants (15.6%) were not certain whether their status had been disclosed (or not) without their consent by health care professionals. Approximately two thirds (75.9%) of participants believed that their records were kept completely confidential.

Other PLHIV, social workers and healthcare workers were perceived as being the most supportive in their reactions when disclosed to, while the highest levels of perceived discriminatory reactions were from teachers, friends and neighbours, spouses/partners, co-workers and employers. A large percentage of participants (354 out of 439 respondents, or 80.6%) indicated that they found their disclosure of their HIV status an empowering experience.

### HIV counselling and testing

Most participants (87.0%) had received both pre- and post-HIV test counselling. More participants (17.9%) that had been living longer with HIV for ten years or more had not received any form of counselling. They were also less likely to have received both pre- and post-test counselling than those living with HIV for less than ten years (76.9% vs 91.4%; *χ*^*2*^=27.634, p<0.001). Among the participants that had been living with HIV for less than ten years, those in Gauteng and Mpumalanga provinces were more likely to have received both pre- and post-test counselling compared to those living in Limpopo or North West (95.5% vs 84.9%; *χ*^*2*^=20.780, p=0.001).

### Access to HIV treatment and care

As would be expected in a sample of patients drawn from an HIV clinic population, a high proportion (87%) of participants were taking antiretroviral therapy (ART), or had access to it even if not taking it (82.5%) at the time of participating in the study, while 59.1% were taking medicine to prevent opportunistic infections. Regarding constructive discussions with healthcare workers in the past 12 months, 69.1% of participants indicated that they had such a discussion regarding their treatment, and 58.8% on other subjects such as sexual and reproductive matters, emotional well-being and drug use.

Confidentiality (44.5%) was identified as the most common challenge experienced during the testing and diagnosis phase, followed by counselling (30.2%), accessibility (15.4%) and resources (11.3%). With regards to challenges in disclosure and confidentiality, 57% identified stigma, 34.2% discrimination and 22% counselling as obstacles. The most common perceived challenge with regard to anti-retroviral therapy (ART) was that participants had to be prepared to take ART life-long (57.8%), followed by side effects (44.3%), monitoring (11.7%) and access (8.2%).

### Having children

Most participants (83.1%) have children, of these an estimated 27.8% of children are known to be HIV positive. Three-hundred and forty-four out of 464 (74.1%) participants indicated that they had previously received counselling about their reproductive options, while 23% were advised by a healthcare worker not to have a child since they were diagnosed with HIV.

The percentage of participants who indicated that they were coerced into being sterilised was 7.1%. About a third (34.2%) stated that their ability to obtain ART was dependent on them using certain forms of contraception and 10/280 (3.6%) indicated that they were coerced into termination of pregnancy in the past 12 months. A high proportion of participants felt that they were being coerced regarding method of delivery (90/277; 32.5) and infant feeding practices (103/278; 37.1%). ART to prevent mother-to-child transmission of HIV during pregnancy was provided to 39.5% of the female participants, while 51.5% indicated that they were not HIV positive during pregnancy. Of those females that did receive anti-retroviral treatment during pregnancy, a majority of 97.4% received some information about healthy pregnancy and motherhood.

### Main problems and challenges experienced by PLHIV

Findings suggest that confidentiality (44.4%) is the most prevalent problem or challenge experienced by participants during the testing and diagnosis phase, followed by counselling (30.3%). Somewhat less problems were experienced in terms of accessibility and resources, ranging between 15.5% and 11.3% of participants. Further findings suggest that a relatively large group of participants (57%) experienced stigma and discrimination (34.2%) in terms of disclosing their HIV status. In contrast, only 6.4% of participants experienced problems in this regard from a healthcare worker perspective. Of concern is the 22.1% of participants that view counselling as a problem in terms of disclosure and confidentiality. The most prevalent problem expressed with regard to ART is the fact that participants must be prepared to take them life-long (57.9%) and the side effects of such treatment (44.3%). Around a tenth (11.8%) of participants experience some problems in terms of monitoring (for example their blood tests) and access to ART. The greatest problem with regard to having children is taking care of them if something happened to the caregiver (43.7%), followed by preventing mother-to-child transmission and the stigma attached to it, 25.4% and 24.2%, respectively, while 12.4% of the participants experience problems in terms of breastfeeding/bottle-feeding.

## Discussion

PLHIV experience stigma - both internalised and externalised - that impact on their ability to optimally make use of intervention services [7,10]. Strategies to address HIV related stigma are not well understood, which impacts negatively on programme planning and service delivery. In order to provide effective services that take into account the realties that PLHIV face, a better informed and evidence-based understanding of stigma is required. Consequently, the current study was performed to broaden the understanding of the extent and forms of stigma and discrimination faced by PLHIV in FPD-supported clinics.

Consistent with other studies, the data from this study demonstrate that PLHIV still face significant stigma and discrimination that impacts across a broad range of aspects in their daily lives [2,5,7,8,26]. Several other studies using the PLHIV Index have been completed, including several in Africa [27-29]. Data from the current study supports the findings from these studies and adds to the international database of information collected using the Index.

### Experiences of stigma and discrimination from other people

More than half of the participants in this study experienced stigma and discrimination following disclosure of their HIV status. This is consistent with data from Kenya, Swaziland and Nigeria obtained in studies using the PLHIV Stigma Index [27-29]. A qualitative study by Cloete et al. [30] also cited their participants’ difficulty in disclosing their status for fear of rejection and concerns with regard to confidentiality. In another study undertaken in South African communities, results indicate that participants who held traditional beliefs about the causes of AIDS were more likely to stigmatise PLHIV [31]. Similar experiences of discrimination and HIV/AIDS-related stigma were reported in a study conducted in an urban informal settlement in Cape Town [32]. Manifestations of such discrimination were furthermore discerned in the first nationally based HIV prevalence study in South Africa, in which 26% of participants revealed that they were unwilling to share a meal with an HIV-positive person, while nearly 18% indicated that they would not sleep in the same room as someone with AIDS [12].

With regards to disclosure, the closer the relationship, the more willing participants were to inform others about their HIV status themselves. Other PLHIV were considered the least discriminatory in their reactions followed by social workers and healthcare workers in terms of the reactions to disclosure. Of concern is the relatively high level of discriminatory reactions from teachers, spouses friends and neighbours, and work colleagues. In the case of teachers, participants reported that they behaved in a manner that discriminated against PLWHIV, this is reported to be a problem in over a fifth of the cases who answered this question (23/104). Discriminatory reactions towards PLHIV upon disclosure should be a key area of focus for educational campaigns and intervention programmes. Surprisingly, especially in light of their relatively high education level, participants reported that teachers relatively often behaved in a manner that discriminated against PLHIV (this was reported to be a problem in over a fifth (23/104) of the cases who answered this question. This is an avenue that needs to be to explored in future research. A large percentage of participants indicated that they have found their disclosure of their HIV status as an empowering experience, these findings are similar to the outcomes of studies that were undertaken in Kenya, Swaziland and Nigeria [27-29].

### Internalised stigma

Similarly to the study of Lee et al. [33] many participants experienced internalised stigma related to their HIV status. This finding is also consistent with results from a study in Kenya using the PLHIV Stigma Index [27]. An important observation of the study is the large differences that exist between the levels of internalised and externalised stigma/discrimination experienced by participants. It is clear that the level of experience of most types of internalised stigma is much higher than levels of external stigma and discrimination experienced (either from others or at work, health services or educational institutions).

Some of the consequences of internalised stigma include those that decided not to have more children because of their HIV status, and others deciding not to get married or to have sex. In terms of the health domain a large number of people opted to avoid going to clinics when needed, while some even avoid going to hospital. This could have a dramatic impact on their treatment and resultant quality of life. In terms of internalised stigma and income generation (wealth domain); just less than one tenth of participants had stopped working, while others did not apply for jobs or promotion, which again could have a significant impact on quality of life.

### Access to work, health and education services

The findings from this study demonstrate that stigma and discrimination experienced by PLHIV negatively impact on their working and family life, as well as their access to education and health services. Participants in this study were in general very poor, with high unemployment rates and more than half of households having a monthly income of <R3000.00. The complex links between poverty, gender power relations, intimate partner violence, drug use and HIV/AIDS have been highlighted in a number of international and local South African studies [10,34-38]. In a study conducted in a black township in Cape Town, findings revealed that AIDS was only one of many major social stressors threatening people living in everyday poverty [39].

Consistent with the data from Stewart and Pulerwitz [40] demonstrating that in the workplace employees suffer from HIV/AIDS-related stigma from their co-workers and supervisors (such as social isolation and ridicule), or experience discriminatory practices (such as being fired from their jobs due to their HIV status); PLHIV participating in this study experienced stigma and discrimination in the workplace from both their employers and co-workers, similar to findings reported by Wolitski et al. [41]. Females were also more likely to experience refusal of employment or work opportunities due to their HIV/AIDS status, as compared to men, findings similar to the results of the study by Hubert et al. [15] which indicated that women are subjected to more HIV- related stigma than men [17]. Possible activities to promote workplace stigma-reduction include training for managers, peer educators, and counsellors, and devising strategies to address stigma and discrimination. As gender differences are also important in the workplace in terms of refusal of employment or work opportunities, workplace programmes should emphasise the importance of reducing blame directed to women.

A range of reasons were provided as to why participants were tested for their HIV status, with the most common reason being that they just wanted to know their status. Encouragingly, the decision to be tested was mostly voluntarily, while few felt under pressure to do so. Furthermore, very few participants were tested without their knowledge or were coerced into it. The majority of participants had received both pre- and post HIV test counselling, while relatively few received only pre-test counselling or only post-test counselling. It is clear that the participants that have been living longer with HIV to a lesser extent have received pre-post counselling, and that they to a larger extent have not received any counselling. This indicates improvements made in availability of counselling services in South Africa. Confidentiality was the most prevalent problem or challenge expressed during the testing and diagnosis phase of HIV, followed by counselling, this data is similar to that obtained for other studies using the Index [27-29].

The relatively high proportion of participants who indicated that they were satisfied with their health at the time of participation in the study, despite their HIV status, is an encouraging finding of the study. This was also demonstrated in the Kenyan study using the PLHIV Stigma Index [27]. More than 85% of participants indicated that they were currently taking ART. However, as the study was conducted amongst PLHIV already attending an HIV clinic, this might not be applicable to a wider population. As PLHIV now live longer and healthier lives due to the greater availability of ART, the urgency of including behaviour change strategies into the public health system becomes an imperative to curb the further spread of the disease, as corroborated in the study of Wolitski et al. [41].

Similar to other PLHIV Stigma Index studies conducted in Africa, the majority of participants indicated that they had had constructive discussions with healthcare professionals regarding HIV treatment [27,29]. However, fewer participants reported having had discussions with healthcare professionals on issues such as sexual and reproductive health, sexual partnerships and emotional well-being; highlighting the need for healthcare professionals to develop an integrated approach when treating HIV positive persons.

### Having children

Since being diagnosed with HIV, approximately three quarters of participants had been advised by a professional not to have a child, and ART to prevent mother-to-child transmission of HIV during pregnancy was provided to less than half of the participants. However, as some of these events may have happened several years ago before the current policies on prevention of mother to child transmission were implemented, it is difficult to interpret the data in the context of current policies and practices. It is encouraging, that of those females that did receive ARV treatment during pregnancy, almost everybody received some information about healthy pregnancy and motherhood. Of more concern is that a number of participants felt that they were coerced into having a termination of pregnancy, being sterilised or using contraception in the last 12 months. Similarly, a large number of participants felt coerced with regards to method of delivery and infant feeding practices.

These findings are of significant concern, as the international human rights framework requires that PLHIV are enabled to fulfil their sexual and reproductive health needs and aspirations. Denying them their reproductive rights is therefore a violation of basic human rights. However, protecting the sexual and reproductive rights of PLHIV remains an area of concern globally and there is increasing recognition that this is an area that requires urgent attention [42]. In the South African context, this indicates a need for education campaigns highlighting the sexual and reproductive rights of PLHIV and directed at both healthcare workers and PLHIV.

### Knowledge of rights, laws and policies and effecting change

It is encouraging that only a relatively small percentage of participants were forced to do things against their rights. The most common violation of human rights was that of being forced to submit to a medical/healthcare procedure against their will, while some people were denied health insurance. Of concern is that just more than half of participants who believe that their rights as a PLHIV have been abused sought legal redress. In comparison, participants in the Kenyan study reported that of the 40% (n=394) who reported that their rights had been abused in the past 12 months, nearly 60% had attempted to seek legal redress; while in Nigeria, 7% (n=260) had attempted to secure legal redress [27,29]. Ways to improve this situation through education campaigns should be investigated. The perceived power of PLHIV to influence policy tended to be much lower for national compared to local issues. It is, therefore, important to ensure PLHIV are aware of existing national policies, and to establish more opportunities for PLHIV to participate at national level.

Approximately half of the participants had confronted, challenged or educated someone who stigmatised and/or discriminated against them. Just less than two-thirds of the participants knew some organisation or group that could assist them if they experience stigma or discrimination from others. It is clear that HIV support groups are the most commonly known organisations, with an overwhelming majority of participants being aware of these groups. In contrast, just less than a fifth of the participants were aware of networking groups However, alhough participants tended to be aware of these some of these groups, only about a third had sought help from any of the above groups to resolve issues of stigma and discrimination. The reason for this is unclear and support organisations should seek to address this issue.

With regards to addressing stigma, the following were highlighted by PLHIV; providing support (emotional, physical and referral), educating PLHIV, advocating for the rights of all PLHIV, and public awareness. These aspects should be incorporated in programmes and policies.

### Problems and challenges experienced by PLHIV

Finally, the benefits of the Index, particularly for those conducting it, go further than just collecting this much needed evidence. Ultimately, the Index was intended to serve as a powerful tool to support the collective goals of FPD and its partners; including government, community-based organisations, activists and PLHIV alike to reduce the stigma and discrimination linked to HIV. Although falling outside the tangible scope of this study, it is hoped that the Index fostered change within communities as it was being used.

### Limitations of the study

The study population may not be representative of the wider South African PLHIV population given that (i) other PLHIV population groups were not sampled and only participants from FPD supported ARV sites were recruited, (ii) data relating to the total number of PLHIV is not available, iii) very few participants from ‘traditional’ high-risk groups for HIV such as commercial sex workers, injecting drug users and men who have sex with men were included, iv) most participants were on ART, therefore no meaningful conclusions on the impact of stigma on access to treatment could be drawn, and (v) retrospective answers has potential difficulties associated with it, such as the problem of recall and the possibility that events and circumstances might be reinterpreted or presented in ways that suit the individuals’ current perspective and perception of self. However, the aim of the study was not to provide a basis for substantial generalisation, but rather to provide an explorative and descriptive account of the attitudes of a group of PLHIV at various FPD-supported clinics in different provinces.

Another potential limitation of the study is the aspect of language barriers, which may have adversely influenced the information gathered in the study. In order to minimise this, the Index was translated by linguistic experts into three indigenous languages, and the interviews were conducted by interviewers who spoke the relevant indigenous languages.

## Conclusions

The study demonstrated that PLHIV experience significant levels of stigma and discrimination that negatively impacted on their working and family life, as well as their access to health services. The most important aspects that were highlighted to address stigma related to the provision of support, educating PLHIV, advocating the rights of all PLHIV, and fostering awareness and knowledge amongst the public. The study also shows that it is possible to use the Index to measure the extent of stigma and discrimination as well as that of internalised stigma. More research will however be required to validate such scales.

The findings can ultimately be used to inform programmes and interventions to reduce stigma experienced by PLHIV. It is clear that the current measures for dealing with stigma should be expanded to incorporate the many issues related to health, education and work discrimination that emerged from the study. Interventions could include behaviour change communication programmes to address internalised stigma as well as discriminatory behaviour towards PLHIV. Specific education programmes to emphasise the rights of PLHIV (especially sexual and reproductive rights) should be developed and programmes established to ensure that these rights are not violated. Finally, efforts should be made to ensure PLHIV are not only aware of their rights, but are empowered to seek redress if these rights are violated. A potential solution could be the introduction of PLHIV ‘champions’ at various service points such as ARV clinics and sexual health and reproductive clinics.

## Competing interests

The authors declare that they have no competing interests.

## Authors’ contributions

MMLDS drafted the original manuscript and assisted with the study conceptualisation, fieldwork training, and data interpretation. PK provided statistical analysis, interpretation of the data, and commentary on the technical quality of the paper. SM assisted with the conceptualisation of the study and the training of fieldworkers. GW sourced the funding for the study, advised during study conceptualisation and provided input relating to the technical quality of the paper. EVDR assisted with the training of fieldworkers, data interpretation and technical quality of the paper. All authors, MMLDS, PK, SM, GW and EVDR, have read and approved the final manuscript.

## Authors’ information

Monika dos Santos holds a PhD in psychology and is a senior lecturer in the Psychology Department at the University of South Africa. Pieter Kruger holds a PhD in psychology and is an associate professor in the Psychology Department at the University of South Africa. Shaun Mellors has been living with HIV since 1986, he is a HIV/AIDS community activist and Associate Director: Africa at the International HIV/AIDS Alliance based in the United Kingdom. Gustaaf Wolvaardt is the founding Managing Director of FPD, and a medical specialist (internal medicine) by training. Prior to this he was South Africa’s first Health Attaché based at the South African Permanent Mission in Geneva where he re-established technical cooperation between the South African health sector and the international community in Europe as South Africa moved to democracy. Elna van der Ryst holds both MD and PhD qualifications and is an experienced medical virologist working as an independent consultant and based in the United Kingdom. She was involved in this study during her Pfizer Global Health fellowship at FPD.

## Pre-publication history

The pre-publication history for this paper can be accessed here:

http://www.biomedcentral.com/1471-2458/14/80/prepub
